# Fluid Degradation Measurement Based on a Dual Coil Frequency Response Analysis

**DOI:** 10.3390/s20154155

**Published:** 2020-07-26

**Authors:** Jose M. Guerrero, Alejandro E. Castilla, José Ángel Sánchez-Fernández, Carlos A. Platero

**Affiliations:** 1 Department of Automatic Control, Electrical and Electronic Engineering and Industrial Informatics, Universidad Politécnica de Madrid, E-28006 Madrid, Spain; josemanuel.guerrero@upm.es (J.M.G.); alejandro.enfedaque.castilla@alumnos.upm.es (A.E.C.); 2 Department of Hydraulic, Energy and Environmental Engineering, Universidad Politécnica de Madrid, E-28040 Madrid, Spain; joseangel.sanchez@upm.es

**Keywords:** degradation, fault diagnosis, frequency response analysis, predictive maintenance, oil insulation, capacitive sensors, magnetic sensors

## Abstract

Electrical industry uses oils for cooling and insulation of several machines, such as power transformers. In addition, it uses water for cooling some synchronous generators. To avoid malfunctions in these assets, fluid quality should be preserved. To contribute to this aim, a sensor that detects changes in fluid composition is presented. The designed sensor is like a single-phase transformer whose magnetic core is the fluid whose properties will be measured. The response of this device to a frequency sweep is recorded. Through a comparison between any measurement and a reference one corresponding to a healthy state, pollutants presence, such as water in oil or salt in water, can be measured. The performance of the sensor was analyzed through simulation. In addition, a prototype was built and tested measuring water concentration in oil and salt content in water. The correlation between pollutant concentration measured with the sensor and known pollutant concentrations is good.

## 1. Introduction

Fluid degradation is responsible for numerous equipment failures. Since the use of fluids in industrial processes is extended to practically every area [1], its degradation is one of the most important parameters to control in an industrial installation.

In many installations, fluids are used to evacuate the heat of an industrial process. For this reason, they are exposed to high temperatures that can cause the breakup of their molecules or at least their cracking [2]. This causes an alteration in the characteristics of the fluid [3] and, therefore, a significant reduction in the performance and efficiency of the industrial processes in what they are applied to.

Another problem resulting from fluids degradation is their oxidation [4]. Chemical oxidation reactions take place when a fluid is exposed to air. This oxidation causes an increase in its viscosity [5]. It can even generate sediments [6,7], with the obvious damage the sediments can cause as they are deposited on duct walls or interact with the fluid.

In the electrical field, oils are the fluids most widely used to insulate the conductors in high voltage application machines as well as used as a cooler, e.g., in power transformers [8]. In this case, the degradation of oil is mainly caused by the cellulose decomposition in water, which causes a lack of insulation properties in the oil [9].

Due to the fluid degradation issues mentioned above, continuous monitoring is very useful to know the fluid condition at all times. This knowledge allows the timely removal of the different components formed by fluid degradation through techniques such as decantation or distillation or, in situations with an advanced degradation, replacing the fluid with a new one to avoid possible future failures [10].

There are several measurement techniques currently used to evaluate fluid degradation [11,12]. They can be classified as chemical and electrical. These techniques can be complemented by some intelligent techniques to draw a conclusion about the degradation degree of the fluid under consideration. Although some other techniques have been proposed, they have not gained momentum [13].

Among the chemical techniques, dissolved gas analysis (DGA) is the most used [14,15]. It is usually accomplished through chromatography [16]. There are several methods to draw conclusions from a DGA. Most of them are based on ratios of different compound concentrations, such as the Doernenberg or the Rogers’ method. The Duval’s triangle and pentagons combine several ratios to achieve a diagnostic about transformer oil degradation [17,18]. Another technique used to evaluate fluid degradation is ultraviolet spectrophotometry [19]. It is based on determining the absorbance spectra of a fluid sample.

Regarding electrical techniques, they can be classified as time domain and frequency domain [11]. These techniques analyze the dielectric response of the fluids. The polarization and depolarization current (PDC) and the recovery voltage (RV) measurements are time-domain based techniques [20].

The PDC method consists of applying a DC charging voltage to a sample and measuring the polarization current. Then, after removing the DC source, the sample is short-circuited, and the depolarization current is measured [21].

The RV method has a first part similar to the PDC method, i.e., applying a DC voltage to a sample. However, in the RV method, the voltage magnitude applied is measured. Then, the DC source is also removed, and the sample is also short-circuited. After a defined discharging period, the short circuit is cleared, and the remaining voltage is measured [22].

Frequency domain spectroscopy (FDS) consists in measuring the dielectric dissipation factor or loss factor of a sample [23]. From the electrical methods, fluid conductivity can be deduced [24]. It should be taken into account that the values obtained are temperature dependent. Therefore, this parameter should be constant to make comparisons between healthy and degraded fluid samples.

Finally, some methods based on frequency response analysis (FRA) are used to diagnosis the state of electrical machines, such as power transformers [25]. FRA makes a frequency sweep injecting a fixed amplitude voltage, usually 1 V or 10 V (root mean square or RMS value), in terminals of the transformer, and analyzes the transfer function between its terminals. Depending on the state of the insulating fluid, i.e., the oil, the transfer function will differ depending on water concentration or temperature. Additionally, impedance polar plots are used [25]. One of the drawbacks of this method is the necessity of stopping the operation to perform the test. 

Online technologies for fluid degradation include the measurement of the complex permittivity produced by the insulator fluid among electrodes used as sensors, which changes with an increase in pollutant agents [26].

This research focuses on the design of a new sensor for measuring fluid degradation based on frequency response analysis. This sensor has two coils immersed in the fluid under test. Attending to the gain variation within a defined frequency range, the concentration of pollutant agents can be determined.

The paper is structured as follows: Section 2 explains the design of the proposed sensor. Then, Section 3 describes a simulation model of the sensor, and Section 4 reports the experimental measurements made to validate the designed sensor. Finally, Section 5 presents the main contribution of the paper.

## 2. Materials and Methods 

**Figure 1:**
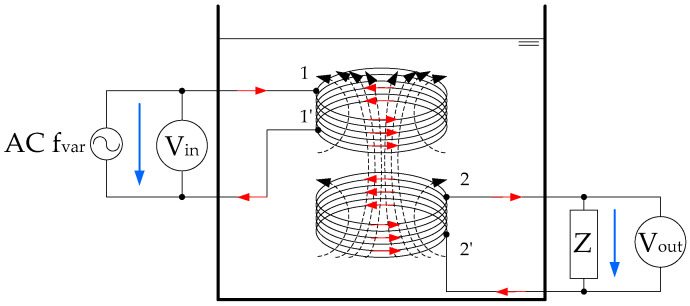
Fluid core dual-coil sensor, magnetic flux scheme.

The proposed sensor is like a transformer where its windings are two coils, and its magnetic core is the fluid under test, as shown in Figure 1. The voltage applied to the first coil causes the magnetic flux common to both coils. This magnetic flux induces a voltage in the second coil. This induced voltage is measured by the FRA test equipment.

**Figure 2:**
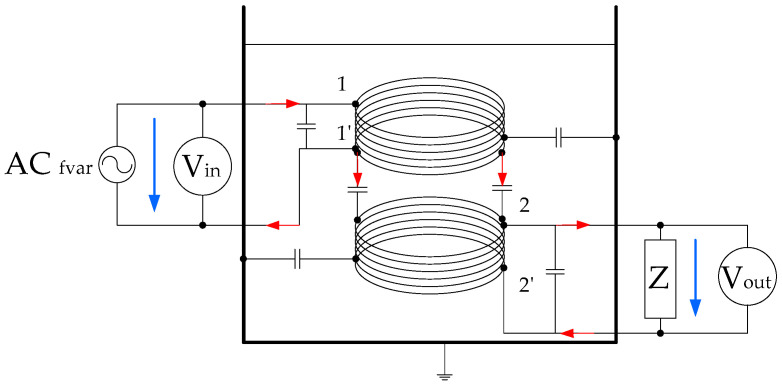
Frequency response analysis (FRA) test fluid core dual-coil sensor capacity couplings simplified circuit.

Both windings are also capacitively coupled. This capacitive coupling is increasingly important as the frequency of the applied voltage increases. The simplified equivalent circuit is displayed in Figure 2. The increase or decrease in fluid permittivity implies higher or lower leakage currents in the system, as can be seen in the scheme proposed in Figure 2.

**Figure 3:**
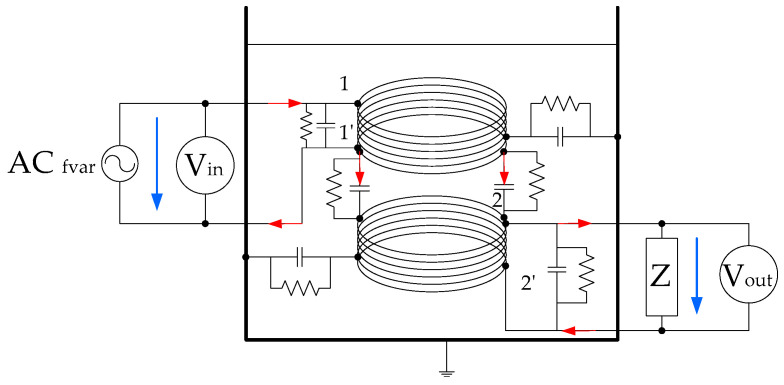
FRA test fluid core dual-coil sensor simplified circuit.

And finally, the effect of fluid resistivity (*ρ*) modifies the energy losses in these couplings. The insulation resistance to ground, between the windings and between turns is modified in case of fluid degradation. The simplified circuit of the fluid core dual coil is shown in Figure 3.

**Figure 4:**
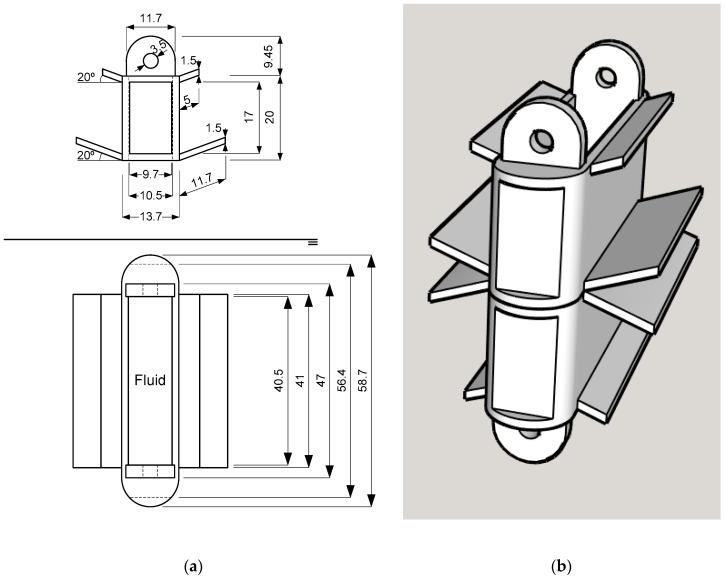
Dual coil holders’ design (**a**) Fluid core dual coil holder dimensions. (**b**) Dual coil holders’ disposition. They were aligned vertically for the free fluid circulation between them.

This phenomenon is quite difficult to characterize due to the different compounds present in a fluid [27]. The interactions among these compounds can cause resistance changes. The variations in conductivity can be linear functions, such as in H_2_SO_4_–H_2_O electrolytes, logarithmic functions, as in NaCl–H_2_O electrolytes, etc., which makes its characterization difficult. The design of the fluid core dual-coil sensor prototype is shown in Figure 4a. A coil was placed on each holder. These coils had N = 200 turns each, with a circular conductor section of S = 0.2 mm^2^ and 0.504 mm diameter. The two holders must be placed vertically and an empty cavity between them. This cavity was filled with fluid when the sensor was submerged in it. The number of turns was 200 to keep the sensor compact but with high enough resolution to observe fluid behavior.

This geometry allowed the fluid to circulate between both coils. A 3D representation of the two coil holders, one for each wiring confronted in a vertical disposition, is pictured in Figure 4b. This disposition allowed the free fluid circulation into the core column.

**Figure 5:**
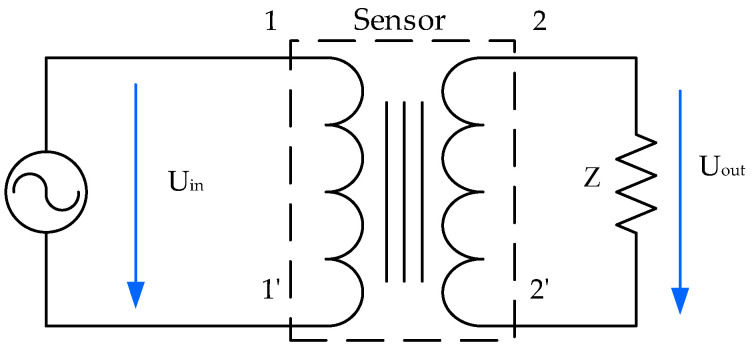
Frequency response analysis test equipment connections in the fluid core double coil sensor.

Once the double coil was submerged in the fluid recipient and this fluid surrounded the sensor, a frequency response analyzer (FRA) test equipment was connected between the terminals of each coil, as shown in Figure 5. The input voltage (U_in_) was applied between 1 and 1’ terminals of one coil. The amplitude was 1 V, and the frequency went from 20 Hz to 20 MHz. The voltage in the other coil (U_out_) was measured by the FRA test equipment. The input impedance of the FRA test equipment was normally a 50 Ω resistor.

The FRA test consisted of contrasting the input with the output voltage in the different frequencies range as (1):

(1) $$A\left(dB\right)=20\cdot \log\left(\frac{U_{\mathrm{out}}}{U_{\mathrm{in}}}\right)$$

If the composition of the fluid changes, then the FRA test results will also change.

To sum up, the Frequency Response Analysis test results, in general, depend on several factors related to the fluid as magnetic permeability (*µ*), permittivity (*ε*), and resistivity (*ρ*). In the case of fluid degradation, a change in its composition occurs, and, therefore, the FRA test results would change. However, it should be noted that, in the cases presented (water in oil and salt in water), there are only changes in resistivity.

The inductive coupling will be significant in the low-frequency range. However, the capacitive coupling of the windings will have more importance at the high-frequency range. As the capacitance increases, the impedance between conductors and ground decreases, while the voltage drop in the system is lower, and the gain response gets higher.

Finally, if water is added to an insulating fluid, the resistivity should decrease, making this fluid a better conductor for leakage currents. This implies a decrease in fluid resistance and, by consequence, a lower voltage drop in the transfer function. For this reason, the gain obtained by registering U_out_ will be higher.

## 3. Simulations

**Figure 6:**
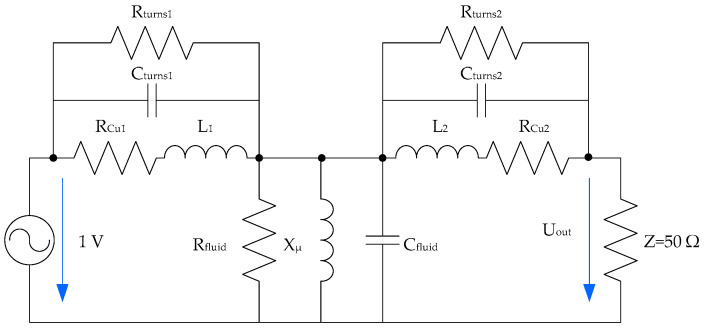
Electrical equivalent circuit used for simulations.

To make simulations, a model of the proposed sensor was needed. This model is shown in Figure 6. The calculation process consisted of solving this circuit for different frequencies between 20 Hz and 20 MHz. The individual calculation took into account the circuit parameters, the variable frequency voltage source, and the voltmeter impedance. Therefore, the U_out_ was obtained, and circuit gain could be calculated, as expressed in Equation (1).

The model was based on the traditional equivalent circuit of a transformer, but included the capacitive couplings and insulation resistances. Models similar to the one presented here can be found in the literature [28,29,30].

The parameters of the equivalent circuit were calculated, attending to the geometrical coil disposition and materials, using well-known formulae. Later, they were fine-tuned, attending to the initial conditions of an FRA test in the unpolluted main fluid. 

The parameters of the equivalent circuit were:

The coils resistances R_Cu1_ and R_Cu2_ were calculated from copper resistivity, wire section, length, and number of turns, as can be seen in Equation (2):

(2) $$R_{\mathrm{Cu}}=\rho_{Cu}\cdot \frac{N\cdot l_{\mathrm{Cu}}}{S_{\mathrm{Cu}}}$$

where l_Cu_ was, according to Figure 4, 0.167 m, the wire section (S_Cu_) was 0.2 mm^2^, and the resistivity at 25 °C was 0.0171 Ω·mm^2^/m.

The initial values for the induction L_1_ and L_2_ were calculated as in (3):

(3) $$L_{1}=L_{2}=\frac{N^{2}}{\frac{1}{\mu }\cdot \frac{l}{S}}$$

where *l* was the average length of the magnetic circuit: 0.0867 m (2 times the length of the sensor limbs and the top and bottom yokes), and *S* was the section of the empty window of the sensor where the fluid circulates (3.93 × 10^−4^ m^2^).

Additionally, the capacitance between the sensor to the ground was estimated as the interaction between two equivalent wires of diameter, *d*, 0.504 mm in the middle of each coil, and using a relative electrical permittivity of 80.5, which corresponded to distillated water. Then (4) can be expressed as:

(4) $$C_{\mathrm{fluid}}=\frac{\varepsilon_{r}\varepsilon_{o}}{\ln\frac{D_{\mathrm{Cu}1\mathrm{Cu}2}}{d}}$$

where D_Cu1Cu2_ was the distance between equivalent wires.

The capacitance between turns has been calculated as the capacitance between two adjacent wires 2 μm apart and using the known expression below, (5):

(5) $$C_{\mathrm{turns}}=\frac{\varepsilon_{r}\varepsilon_{o}}{\ln\frac{2d+2\;\left[\mu m\right]}{d}}$$

On the other hand, the resistances between turns (R_turns1_, R_turns2_) and between coils were considered through the fluid (R_fluid_). So, they were calculated as a direct multiplication between the fluid electrical resistivity (distillated water = 3 MΩ/cm) and the insulation length between cables or between equivalent cables. The magnetizing induction was initially considered 5 times lower than L_1_ and L_2_ to provoke high magnetizing currents.

Finally, the parameters were adjusted, taking as reference the response to a test of the sensor submerged in a healthy fluid. The adjusted parameters are shown in Table 1. 

To verify the proposed methodology, circa 200 simulations were performed based on the already described equivalent circuit varying R_turns1_, R_turns2_, C_turns1_, C_turns2_, L_μ_, R_fluid_ and C_fluid_ to achieve the healthy-state fluid transfer function. The fitting process was done by comparing the simulation with an experimental healthy-state salt in the water test, taking as reference the gain value in the plateau (between 200 Hz and 10 kHz) and the first resonance gain (between 200 and 500 kHz), in dB. As shown in Table 1, the impedances of the equivalent circuit of the proposed sensor were different from the usual ones of iron core transformers.

**Figure 7:**
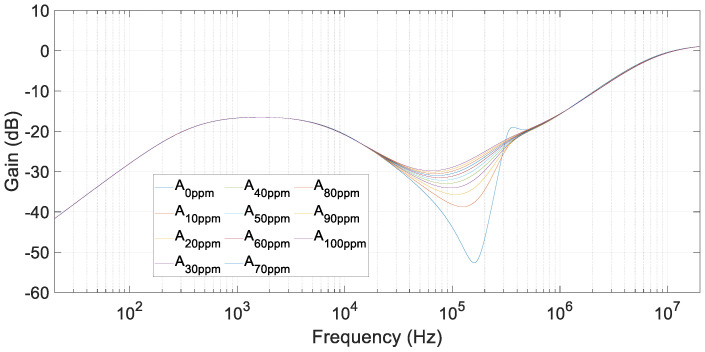
Simulation results for different salt in water concentrations, from 0 ppm up to 100 ppm.

The simulations were performed, varying different parameters of the equivalent circuit. They took into account variations of the core-magnetizing inductance, fluid resistance, and capacitance with the addition of component B into a fluid A. The simulations corresponded to the case of water polluted with salt. Therefore, different salt (component B) quantities were added to water (main fluid). Increasing the frequency from 20 Hz to 20 MHz, emulating the sweeping stages of FRA equipment, the following results were achieved varying the fluid resistance, inductance, and capacitance for salt additions of 0, 10, 20, 30 …, up to 100 ppm weight of salt in 1 L of water and registering U_out_ (see Figure 7). Furthermore, the values of the parameters that depend one fluid composition are displayed in Table 2 for concentrations of 10, 20, 50, and 100 ppm of salt in water.

In these simulations, where salt was added to water, the resistance parameter was highly affected by the addition of the pollutant agent. This was caused by the ions that appeared when salt dissolved in distilled water. Then, the variation of R_turns1_ and R_turns2_ were the main factors of the resonant frequency displacement. A lower value of these parameters implies a softer and lower-frequency resonance.

The brine achieved implies an exponential reduction in water resistance or fast growth of the conductivity that increased from 0.5 μS/cm to approximately 5000 μS/cm for a 0.05 mol/L at a standard temperature of 25 °C dissolution according to [27]. The capacitance and inductance had small changes. The capacitance became lower than in healthy conditions because the relative permittivity of a 2 mol/L brine was 55 versus 80.5 of distilled water. However, the differences were extremely low due to the small variations in salt concentrations. The inductive part of the system also remained nearly constant, being salt a diamagnetic material.

Although different concentrations of pollutant produced different results in the FRA tests that lead to different R, L, C values, the minimum peak achieved in every frequency sweep was located in the same frequency range (20 kHz–200 kHz), as shown in Figure 7. This difference between relative minimums allowed the sensor to distinguish the extent to which the polluting agent was affecting the main fluid. To this aim, the FRA results from a polluting agent concentration should be compared to the corresponding healthy state FRA test (0 ppm of salt in water).

As the interaction between fluids alters the frequency response, a database should be created with different fluid contaminations depending on the application of fluid monitored. The simulations were performed for salt in water. Other fluids were not simulated. 

Once the simulations were carried out, some experimental tests in a prototype must be done to validate the proposed sensor. In addition, the parameters that change with contaminations should be determined.

## 4. Experimental Setup

According to the proposed methodology, impurities or degradation symptoms in fluids could be detected by performing an FRA test with the proposed sensor. However, the fluid must be tested previously in reference conditions. This first test is considered a reference test. Future tests should be compared to the reference test to determine if there is any change in fluid composition.

In the first set of tests, some quantities of NaCl were added to water to modify its properties. In the second set of tests, some quantities of water were added to transformer oil, emulating the degradation in a real power transformer.

**Figure 8:**
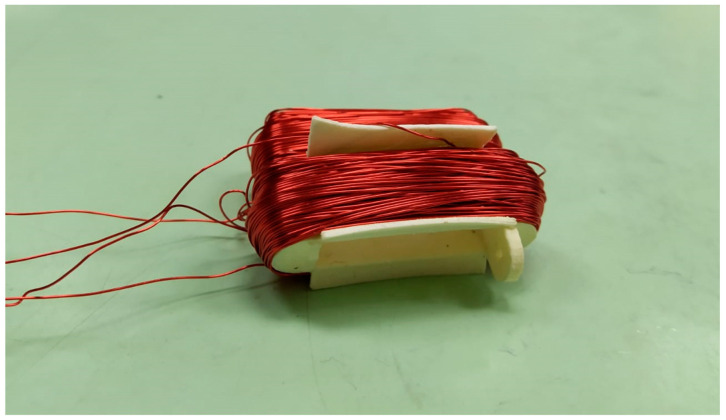
Fluid core double coil sensor used in the experimental setup.

Before the experiments, an experimental setup was prepared. It consisted of the proposed sensor that was going to be submerged in the tested fluid (See Figure 8). In the first coil, a voltage source applied a 1 V_RMS_ sinusoidal wave. The frequency of the voltage source varied from 20 Hz to 20 MHz. The voltage in the second coil was measured and compared to the voltage applied to the first one. The FRA test equipment’s main characteristics are displayed in Table 3.

**Figure 9:**
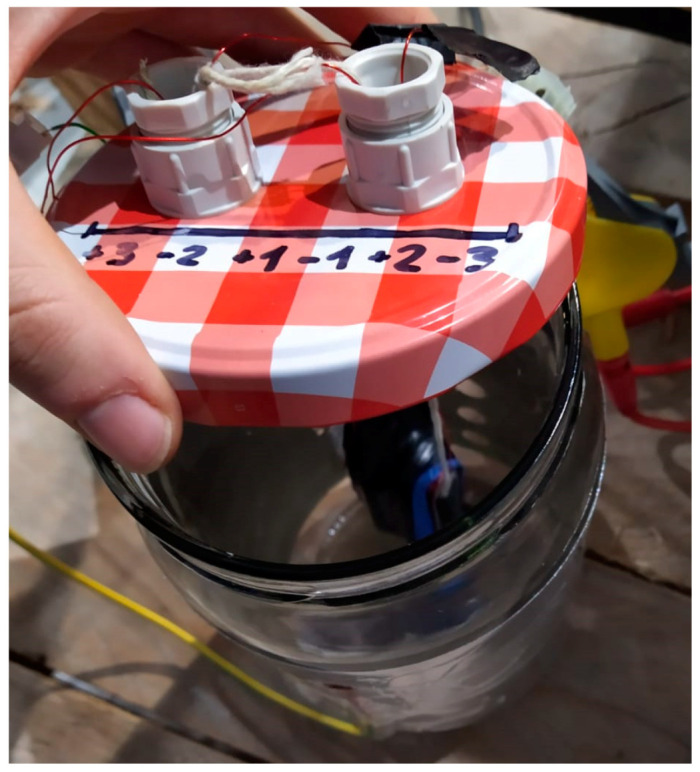
Two-liter container with the fluid core double coil sensor.

Once the connections were done, the sensor was submerged in a two-liter container, as shown in Figure 9. The container was filled with the fluid under test, while a second dissolution was prepared outside with the pollutant agent.

**Figure 10:**
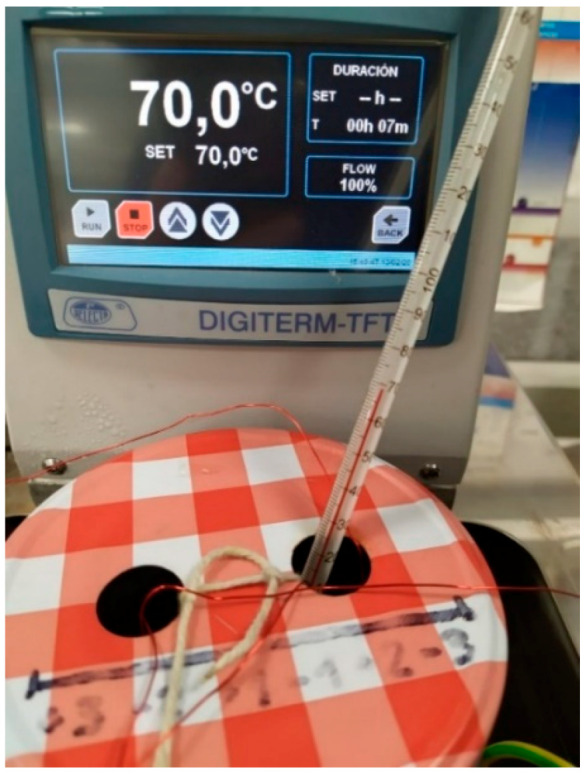
Controlled temperature of the dissolution of oil and water at 70 °C.

**Figure 11:**
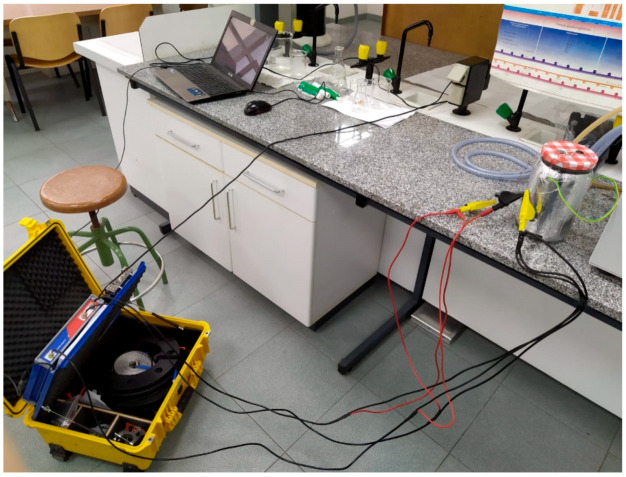
FRA test connections to the two-liter container. No thermostable bath was done in this test.

The tests were performed at constant temperature conditions. The container was placed in a controlled temperature tank (see Figure 10). The temperature of the reference test should be close to the normal condition operating temperature, e.g., oil and water tests, emulating a transformer cellulose decomposition from the insulation materials, were performed at 70 °C. Then, the addition of a second component was done with the aim of carrying out different FRA tests with different pollutant agent concentrations. The FRA connections to the experiment are displayed in Figure 11, where the whole system can be observed (from left to right: FRA Equipment, computer for signal monitoring, and the recipient with the tested fluid). Once the connections were done, the FRA tests were performed, taking around 5 min per test.

As electric conductivity depends on temperature [31], the FRA test results will be affected by operating temperature. Therefore, the temperature of the different tests should be constant. Hence, contrasting the tests and the reference test can be done. In this paper, only one-constant temperature tests were carried out.

### 4.1. Distilled Water Contaminated with Salt Experimental Tests 

Firstly, a set of experiments with pure distilled water with additions of salt was conducted. The tests were performed with 1.7 L of water in a recipient. An additional dissolution of salt in 1 L of water was used to add small ppms of salt to the first recipient. With those recipients, the addition of the brine in the first container was progressive, then only 2.7 L of water and less than 1 g of NaCl were used.

**Figure 12:**
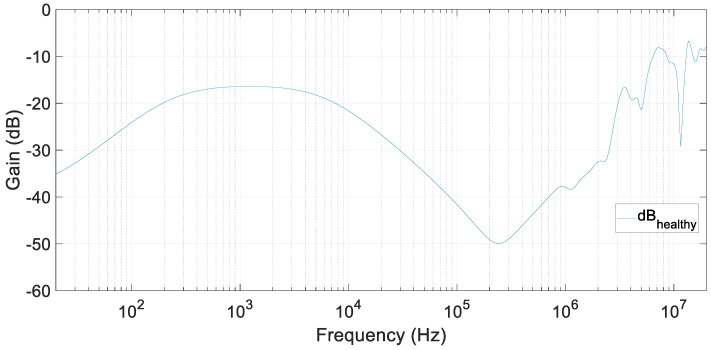
FRA test results with pure distillated water. Reference test.

They consisted of filling the container with pure distilled water and dive the sensor in the container. After this, an FRA test was performed, obtaining the results shown in Figure 12. In this figure, the frequency response of pure distilled water can be seen. This test corresponded to a healthy state of the fluid, and it has been be considered as the reference test. The frequency of test injection equipment went from 20 Hz up to 20 MHz.

**Figure 13:**
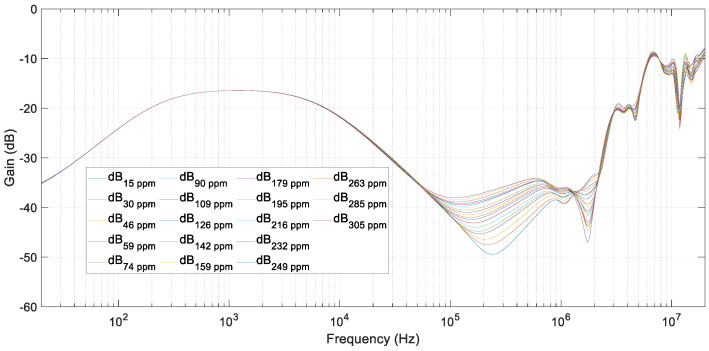
FRA test results from 15 ppm up to 305 ppm of salt in water.

**Figure 14:**
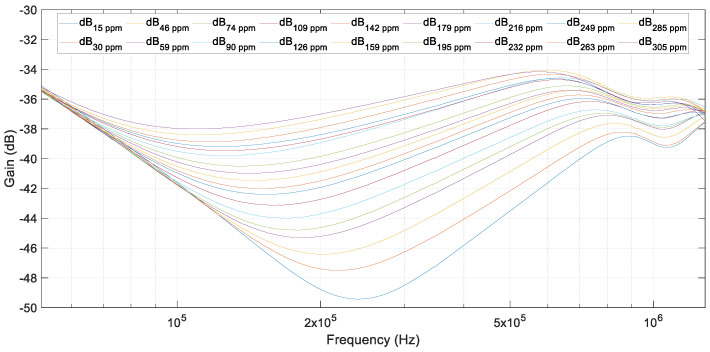
FRA test results from 15 ppm up to 305 ppm of salt in water. Zoom (100 kHz to 500 kHz).

The next step was the addition of salt at an approximate ratio of 15 ppm. The effects in the frequency response can be observed in Figure 13 and Figure 14. In them, the results of tests for salt concentrations varying from 15 ppm to 305 ppm are shown. The FRA results were clearly different depending on the quantity of salt in water. As can be observed, the resonant frequency and amplitude changed in a monotonic way. This is an important fact to determine the quantity of salt in the water. In these experimental tests, a resolution of 15 ppm was corroborated.

**Figure 15:**
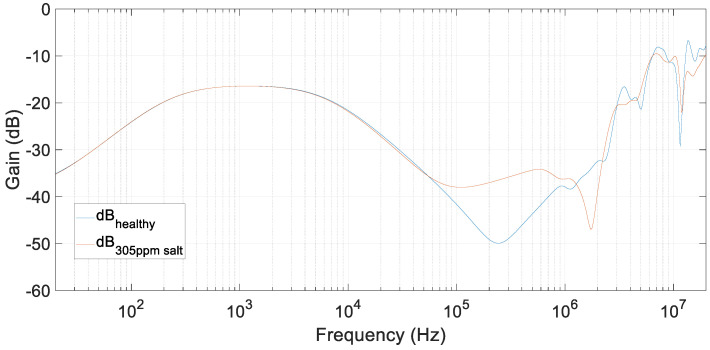
FRA tests for a transformer with distillated water core with salt. Tests compare distilled water and water with 305 ppm of salt.

A comparison of the last test, with water polluted at 305 ppm of salt, and the reference test, with pure distilled water, is presented in Figure 15. It can be clearly observed how the frequency response varied in the range of 100–500 kHz (see Figure 14). However, at low frequencies, impedance remained almost constant, and the FRA results were not influenced by the addition of salt.

In Figure 15, a second resonance appeared that should be provoked by the added salt. When comparing this figure with Figure 13, this second resonant peak did not appear in simulations. This is because this model has been fitted to get an optimum response in the frequency range of up to 500 kHz.

**Figure 16:**
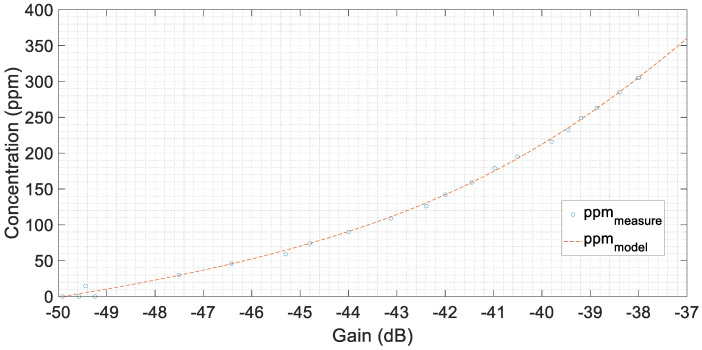
Measures of the minimum value of gains in the range of 100 kHz up to 500 kHz and the fitted curve.

To estimate the salt quantity by the analysis of the FRA results, the gains at the resonant frequency were used. These gains corresponded to the minimum gains in the frequency range from 100 kHz to 500 kHz. The minimum gains vary in a monotonic way, as shown in Figure 16.

Data presented in Figure 16 can be fitted by Equation (6) achieving a coefficient of determination, R^2^, of 0.9987:

(6) $$C\left[\mathrm{ppm}\right]=f\left(A\left[\mathrm{dB}\right]\right)=k_{1}\cdot e^{\alpha \cdot A}+k_{2}\cdot e^{\beta \cdot A}$$

where

$$k_{1}=1.102\times 10^{5}\;;k_{2}=-24.13\;;\;\alpha =0.1512\;;\;\beta =-0.01762$$

These results corroborate the possibility of characterizing water contamination with salt by FRA analysis. 

### 4.2. Oil Contaminated with Water Experimental Tests 

These second laboratory experimental tests aimed to detect water in power transformer oil. During the lifetime of power transformers, insulation degrades, especially at the end of their useful life. 

During this process, water is released from cellulose, which is one of the main components of the insulation system of a power transformer. In addition, water is mixed with oil; therefore, it is essential to monitor the content of water in oil. High concentrations of water can initiate partial discharges and even cause catastrophic failures in power transformers. These phenomena occur when the cellulose of the insulating Kraft paper decomposes. 

In this case, the tests were performed with 1.7 L of oil and an additional dissolution of 1 gram of water in 1 L of oil that allowed the addition of water in 1 or 10 ppm increments to the main recipient.

The objective of these experimental tests was to measure in ppm the concentration of water in oil using FRA tests. 

**Figure 17:**
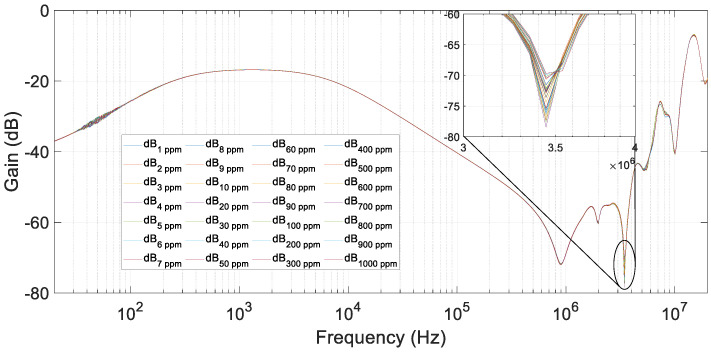
FRA test results for dissolutions of oil and water from 1 to 1000 ppm of water in oil.

Numerous tests with oil and water were performed. The test results are displayed in Figure 17. The procedure was similar to the previous tests. However, now the pollutant agent was water. The tests were carried out from 1 ppm up to 1000 ppm of water in 1.7 L of oil. The water contamination addition steps were 1, 2 … up to 10 ppm, 10, 20 … up to 100 and finally 100, 200 … up to 1000 ppm. 

In these tests, the resonant frequency range went from 3 MHz up to 4 MHz because of the higher insulation capacity and resistance of oil compared with water. In this range, the variation in gain was small but not negligible. Comparing Figure 17 with Figure 15, in oil and water mixtures, the frequency resonance was already present in a healthy state, and water addition smoothed it. However, in salt and water mixtures, the behavior was the opposite.

**Figure 18:**
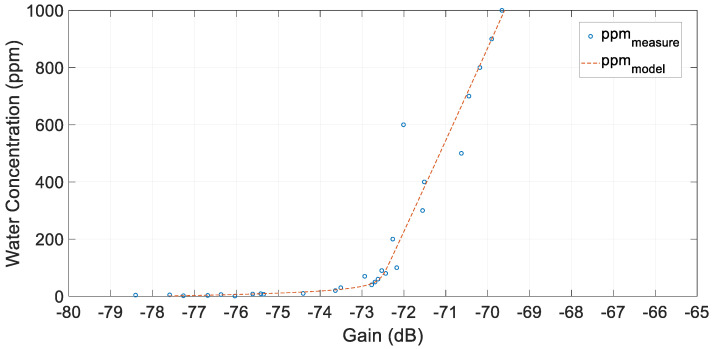
Measures of the minimum value of gain in the range of 3 MHz up to 4 MHz and the fitted curve.

The evidence of how the FRA varied due to the addition of water is shown in Figure 18, where the gain was plotted against the concentration of water in oil. In these experiments, 1 ppm resolution was achieved. To get this sensitivity, a good fluid circulation and homogenization of the mixture was required.

In this case, the most accurate model adjustment was a model of gain from water concentration, as shown in (7)

(7) $$A\left[\mathrm{dB}\right]=f\left(C\left[\mathrm{ppm}\right]\right)=k_{1}^{\prime }\cdot e^{\gamma \cdot C}+k_{2}^{\prime }\cdot e^{\delta \cdot C}$$

Afterward, the concentration could be estimated thanks to a lookup table made from the previous model. In this case, the model parameters were:

$$k_{1}^{\prime }=-5.279\;;k_{2}^{\prime }=-72.72\;;\;\gamma =0.07425\;;\;\delta =-4.391\times 10^{-5}$$

with a regression coefficient of 0.9843.

Due to the low conductivity of the transformer oil, the accuracy of the method was lower than in the previous cases with water and salt. However, thanks to the 3.4 MHz minimum peak, the concentration of water in oil could be calculated.

## 5. Conclusions

Fluid degradation is the cause of failure in many industrial processes or machines. For example, in the field of electrical machines, power transformers and large turbo-generators need pure oil and water, respectively. In power transformers, the oil, used as cooling and insulating medium, should not be contaminated with water. Otherwise, insulation failures can take place with severe consequences. In large generators, the cooling systems comprise pure water to directly cool down the stator windings. This water should have an extremely low conductivity; otherwise, an internal fault could take place.

In the paper, the design of a fluid core double coil sensor prototype was presented. The prototype was manufactured, and numerous experimental tests were performed to detect contamination of pure water with salt and contamination of transformer oil with water. In both cases, it was possible not only to detect contamination but also to estimate the amount of contaminant. For this, the minimum gain in the transfer function, corresponding to the resonant frequency was analyzed. Monotonic behaviors were observed in both cases. Hence, fit curves were calculated. 

In addition, a simulation model was presented. The simulation model was based on the equivalent electric circuit of the double coil sensor. In this case, pure water contaminated with salt was used for the simulations. Numerous simulations were carried out, varying the components of the equivalent electric circuit of the model according to the salt quantity in the water. Similar results to the experimental tests were obtained. 

In this paper, a novel sensor to detect fluid degradation or contamination was presented. The diagnosis technique was based on analyzing the frequency response analysis (FRA) of the sensor immersed in the tested fluid. The sensor comprised two coils with an open core. The first coil was fed at constant voltage with variable frequency (from 20 Hz to 20 MHz), while the voltage in the second coil was recorded. Afterward, its transfer function was calculated, dividing the voltage measured in the second coil with the voltage injected in the first one. The FRA test performed in normal operating conditions was considered as the reference test. The changes in the FRA tests were due to variations in the magnetic permeability, electric permittivity, and resistivity of the fluid. Therefore, if there is a contamination or degradation in the fluid, it can be detected by comparing an FRA test with the FRA reference test.

In terms of future research, the double-coil sensor dived in the power transformer tank must be tested with higher voltages, so as to create higher fluxes that would induce larger voltages in the second coil and improve the sensitivity of the method. Besides, temperature influence will be considered and corrected with experimental parameters to standard ranges. For this, numerous experimental FRA tests must be conducted for different temperatures and pollution sweeps. These improvements can be important for developing a sensor that allows online fluid degradation measurement.

## 6. Patents

As a result of this work, a patent has been applied for, Spanish Patent P202030539 “Sistema y método de medición de degradación o contaminación de fluidos mediante un sensor inductivo de núcleo hueco”.

## Figures and Tables

**Table 1:** Simulation model, initial parameters.

Parameter	Value	Units
R_Cu1_	2.86	Ω
L_1_	9.87 × 10^−4^	H
R_Cu2_	2.86	Ω
L_2_	9.87 × 10^−4^	H
R_turns1_	2000	Ω
C_turns1_	9.05 × 10^−10^	F
R_turns2_	2000	Ω
C_turns2_	9.05 × 10^−10^	F
R_fluid_	2 × 10^7^	Ω
L_μ_	1.97 × 10^−4^	H
C_fluid_	1.63 × 10^−10^	F

**Table 2:** Simulation model, parameters’ evolution when the salt concentration in water varies.

Parameter	10 ppm	20 ppm	50 ppm	100 ppm
L_1_ (H)	9.87 × 10^−4^	9.87 × 10^−4^	9.87 × 10^−4^	9.87 × 10^−4^
L_2_ (H)	9.87 × 10^−4^	9.87 × 10^−4^	9.87 × 10^−4^	9.87 × 10^−4^
R_turns1_ (Ω)	780	612	446	349
C_turns1_ (F)	9.05 × 10^−10^	9.05 × 10^−10^	9.05 × 10^−10^	9.05 × 10^−10^
R_turns2_ (Ω)	780	612	446	349
C_turns2_ (F)	9.05 × 10^−10^	9.05 × 10^−10^	9.05 × 10^−10^	9.05 × 10^−10^
R_fluid_ (Ω)	7.80 × 10^6^	6.12 × 10^6^	4.46 × 10^6^	3.49 × 10^6^
L_μ_ (H)	1.97 × 10^−4^	1.97 × 10^−4^	1.97 × 10^−4^	1.97 × 10^−4^
C_fluid_ (Ω)	1.63 × 10^−10^	1.63 × 10^−10^	1.63 × 10^−10^	1.637 × 10^−10^

**Table 3:** Frequency response analysis (FRA) equipment’s main characteristics.

Parameter	Value
Frequency range	10 Hz–20 MHz
Output Impedance	50 Ω
Accuracy (down to −80 dB)	<0.1 dB
Accuracy (down to −100 dB)	<0.3 dB

## Nomenclature

*A*	Gain
*α*	Mathematical coefficient (salt in water model)
*β*	Mathematical coefficient (salt in water model)
*C*	Pollutant agent concentration
C_fluid_	Fluid capacitance between coils and ground
C_turns1_	Capacitance between turns in first coil
C_turns2_	Capacitance between turns in second coil
*γ*	Mathematical coefficient (water in oil model)
*d*	Wire diameter
D_Cu1Cu2_	Distance between equivalent conductors
*δ*	Mathematical coefficient (water in oil model)
*ε*	Electric permittivity
*k*_1_	Mathematical coefficient (salt in water model)
*k*_2_	Mathematical coefficient (salt in water model)
*k*_1_^′^	Mathematical coefficient (water in oil model)
*k*_2_^′^	Mathematical coefficient (water in oil model)
*l*	Average length of the magnetic circuit
L_1_	First coil inductance
L_2_	Second coil inductance
l_Cu_	Conductor’s length
L_μ_	Magnetizing inductance
N	Turns number
*μ*	Magnetic permeability
R_Cu1_	First coil wire resistance
R_Cu2_	Second coil wire resistance
R_fluid_	Fluid resistance between coils and ground
R_turns1_	Resistance between turns in first coil
R_turns2_	Resistance between turns in second coil
ρ	Electric resistivity
*S*	Magnetic gap section
S_Cu_	Conductor’s section
U_in_	Input voltage
U_out_	Output voltage
Z	Impedance
